# How Do Inorganic Students and Instructors Describe Conductivity in Solids?

**DOI:** 10.1021/acs.jchemed.5c01197

**Published:** 2026-09-09

**Authors:** Barbara A. Reisner, Anne K. Bentley, Joanne L. Stewart, Justin M. Pratt, Shirley Lin, Jessie R. Hallers, Jeffrey R. Raker, Sheila R. Smith

**Affiliations:** § Department of Chemistry & Biochemistry, 3745 James Madison University, Harrisonburg, Virginia 22807, United States; ‡ Department of Chemistry, 6683 Lewis & Clark College, Portland, Oregon 97219 United States; # Department of Chemistry, 3930 Hope College, Holland, Michigan 49423, United States; † Department of Chemistry, 4260 University of Rhode Island, Kingston, Rhode Island 02881, United States; ∥ Department of Chemistry, 32722 United States Naval Academy, Annapolis, Maryland 21402, United States; ⊥ Department of Chemistry, 7831 University of South Florida, Tampa, Florida 33620, United States; ∇ Department of Natural Sciences, 14711 University of Michigan—Dearborn, Dearborn, Michigan 48128, United States

**Keywords:** Second-Year Undergraduate, Upper-Division Undergraduate, Inorganic Chemistry, Scholarship of Teaching and Learning, Conductivity, Metals, Physical Properties, Semiconductors, Solid State Chemistry

## Abstract

This study investigates how inorganic chemistry students and faculty discuss electrical conductivity differences between a metal and a semiconductor using an open-ended question with temperature-dependent conductivity data. The question was deployed across seven foundation-level inorganic chemistry courses in different institutional contexts. Four overarching categories of ideas emerged from the responses: conductivity, doping, band structure, and the effects of temperature. Responses were analyzed on the basis of these categories. The majority of students discussed conductivity and doping, with fewer discussing the band structure and temperature. Students enrolled in courses with a physical chemical prerequisite referenced more ideas, including more submicroscopic ideas, in their responses compared to students in a course that did not have this prerequisite. Instructor responses included ideas from all four categories as well as more ideas derived from submicroscopic models. This pattern is consistent with experts referring to more resources than novices. Generally, these results provide further evidence of the impact that increased exposure and practice have on learning and development of knowledge. These findings reveal that, although students use ideas from the four overarching categories, they do not fully use those concepts to explain the electronic behavior of solids, which suggests the need for instruction that helps students make connections between macroscopic data and submicroscopic explanations. The resources used by inorganic students and suggestions for how these ideas can be surfaced and integrated during instruction are presented herein.

## Graphical abstract

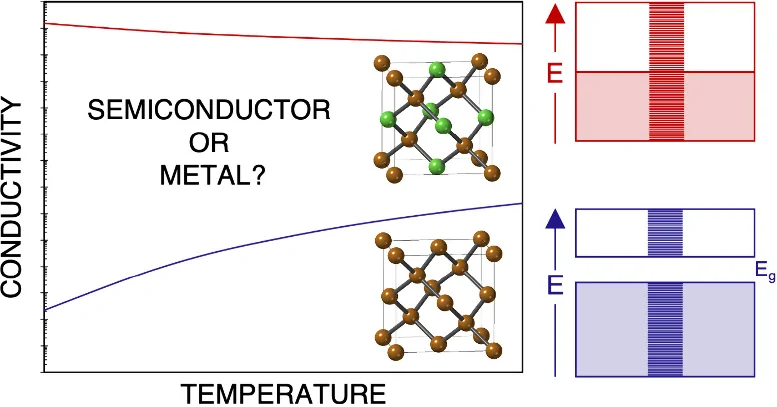

## Introduction

Solids are central to modern technology. For example, the semiconductor silicon is the basis of all modern electronics, lead halide perovskites are being explored as next-generation solar cells because of their tunable band gaps,^1,2^ wide band gap semiconductors play a critical role in renewable energy,^3^ and phosphor materials provide the basis for solid-state lighting.^4–6^ Knowledge of solid-state chemistry, structure-property relationships, and the electronic structure of solids is important for both understanding how materials work and designing the next generation of materials.

While solids and solid-state chemistry are frequently taught in foundation-level inorganic chemistry courses,^7,8^ many instructors that we, the authors, work with report that they do not consider themselves experts on solids and view teaching solids as a weak point. In a 2025 community survey of 168 members of the Interactive Online Network of Inorganic Chemists,^9^ inorganic chemistry instructors indicated that they wanted more support for teaching this topic. As part of the survey, instructors ranked 12 topics where they would like new learning materials created; solids and solid-state chemistry was the most highly requested, tied with spectroscopy and structural methods. Additionally, only one in five instructors surveyed felt that they had the expertise to create instructional materials for teaching solids and solid-state chemistry.

The lack of instructor confidence in solid state chemistry is not surprising given that a faculty member’s research and training in inorganic chemistry spans the range of small molecule chemistry to biological systems; historically, the chemistry curriculum, across all subdisciplines, has focused on small molecule chemistry. Many undergraduate inorganic chemistry courses also aspire to cover the scope of the field, which means that instructors are often tasked with teaching outside of their specific area of expertise.^7,8,10^ Because of the breadth of the field, instructors may not have a good understanding of the limitations of concepts and models that they are teaching, since that understanding comes with advanced study and research experience.^11^

In our study, we investigated student and instructor ideas about electrical conductivity (henceforth termed conductivity) in solids to understand the ideas activated by students and instructors with the goals of informing instruction and improving teaching and learning of the topic. Experts with extensive knowledge are more likely to have a greater number of resources that are better coordinated than novices.^12–14^ While significant research has examined teaching and learning in introductory and large-enrollment courses such as general and organic chemistry, fewer studies address upper-division courses and advanced topics.^15^ Student conceptions of conductivity in solids have not been investigated from a chemical perspective, although studies in physics and engineering education discuss this topic.^16–25^ If we are to help chemistry students build their knowledge frameworks, then an important step is to understand the resources that chemistry students and instructors use to think about conductivity in solids.

Herein, we describe how chemistry students and instructors from seven institutions approach an open-ended problem in which they are presented with conductivity data for two solid materials. We evaluate whether respondents engage with conductivity, doping, band structure, and temperature when describing how the electronic structure of the two solids leads to differences in conductivity. Our study identifies the concepts that students invoke when talking about conductivity. We also describe how course sequencing affects the resources that participants use. We use these findings to provide recommendations for how instructors can engage students in productive conversations about the electronic structure of solids. Ultimately, we hope that this study will help instructors reflect on how they, and their students, think about conductivity in solids and how students’ understanding of the electronic structure of solids can be improved.

## Conductivity in Solids: The Chemistry Curriculum and Prior Research

In a chemistry degree curriculum, students are introduced to electrical conductivity at the macroscopic, submicroscopic, and symbolic levels. A macroscopic treatment of electrical conductivity focuses on current, charge, and voltage, while a submicroscopic treatment invokes models used to describe how submicroscopic electrons and atoms impact the electrical conductivity of a material. There are two dominant submicroscopic models used to describe the electrical conductivity of a material: the Drude (i.e., electron sea) model and band theory. The classical Drude model^26^ describes a metal as a sea of constantly moving electrons bouncing off of heavier, and relatively immobile, positive ions. While the Drude model adequately explains electrical conductivity in metals, it fails to explain conductivity in semiconductors. The quantum mechanical band theory describes electrons via wave functions and quantized energy states. The symbolic treatment of semiconductors includes the use of mathematical relationships to describe electrical conductivity as well as representations showing bands and their relative energies.

Students typically encounter macroscopic descriptions of electrical conductivity prior to a university-level chemistry course. At the university level, conductivity is introduced as a macroscopic property describing materials (e.g., metals are conductive and insulators are nonconductive) in general chemistry. The timing of the introduction of submicroscopic models and symbolic language is highly variable. At the submicroscopic level, a metal is typically described as a material with mobile electrons. While some general chemistry textbooks discuss quantum mechanics-based band models, most U.S. programs do not cover band theory in introductory courses. Chemistry students are most likely to encounter band theory in a foundation-level course on inorganic chemistry. Of the 75% of instructors who address topics related to solids, 84% report covering band structure, 81% report addressing semiconductors, and 47% report covering electrical resistivity and conductivity.^8^ A mathematical treatment of electrical conductivity is rarely addressed in the inorganic course; it is typically covered in introductory physics courses with mathematical treatments of current using Ohm’s Law. It may also be addressed in a physical chemistry course, although only 29% of instructors report that they cover any topics related to solids.^27^ Inclusion of conductivity in textbooks is also highly variable; some texts like Rayner-Canham^28^ only discuss the band theory model, while others like Weller et al.,^29^ Housecroft and Sharpe,^30^ or Miessler et al.^31^ connect this model to temperature-dependent conductivity or resistivity data.

While there are multiple studies about student conceptions of electrical conductivity, such studies investigate the ideas of primary or secondary students or postsecondary students taking introductory coursework, where students are unlikely to have been introduced to band theory.^19,32–37^ These studies indicate that many students struggle with the nature of metallic bonding and the concept of electron flow. For example, students think that metallic bonding is not real bonding, that there is no bonding in metals,^34^ or that conductivity results from the motion of atoms.^19,32^ This is not surprising, since many textbooks and online resources contain misinformation about the conductivity of solids.^38^ Other studies focus on conductivity in electrochemical systems; these studies describe the conceptual difficulties that students have with electrochemistry and the factors that contribute to a lack of understanding about the subject, such as incomplete or incorrect background knowledge, compartmentalization of knowledge, students’ interpretations of language, the use of multiple definitions and models, and the rote application of concepts.^39–45^

Investigations of student conceptions *after* exposure to band theory come from the electrical engineering, computer science, and physics education literature.^16–25^ While chemists treat band theory from a molecular level perspective, physics texts^46^ typically present a highly mathematical, wave-based treatment of electrons, while electrical engineering texts^47^ focus on the behavior of systems as well as a mathematical treatment. Studies in these fields found that students often make incorrect connections because they have an underdeveloped understanding of concepts.^16,18^ Generally, students have trouble connecting macroscopic behavior and submicroscopic explanations: they do not always select the best models, or they use incorrect knowledge or naive ideas to construct explanations. Previous work also shows that teachers with no prior content knowledge developed topic-specific pedagogical content knowledge as they learned and gained confidence in their content knowledge.^23^

These studies, along with the use of the microscopic models of electrical and thermal conduction in solids (METCS) concept inventory,^24,25^ uncovered naive ideas and challenging concepts for physics and engineering students. The ideas and challenges that most closely connect to this work and how conductivity is discussed within the inorganic chemistry curriculum include conductivity, doping, band structure, and temperature.

### Conductivity

Students struggle to explain conductivity.^16,18,21,24^ While the classical Drude model works well for metals, it fails to explain conductivity in semiconductors, because quantum mechanics is required. Students struggle with ideas of delocalization and the wavelike nature of electrons; they have an incomplete understanding of band diagrams and may not consider how conductivity is related to material composition.^18^ They also have difficulty understanding which charges are mobile and immobile in a semiconductor^16,18^ and believe that the introduction of electronic holes creates charged materials.^18^ Students state that current is the movement of only one particular type of charge, either electrons or holes,^21^ and that electrons move in a straight line.^18,22,24^

### Doping

Students also have trouble explaining how doping affects conductivity^21,24^ and struggle with the concept of electronic holes.^16,18,19,21,23,24^ Students do not equate holes with electronic structure but rather think of them as structural vacancies, i.e., missing atoms or ions.^16,19,25^ They believe that dopants change the charge on materials, implying that doped semiconductors are not electrically neutral.^18,19,23,24^

### Band Structure

Students generally have difficulty understanding the band model^17^ and the concept of a band gap.^18^ Even when students have been introduced to this model, they are not likely to use it to explain phenomena.^20,21^ Students are also likely to mix up the terms *valence band* and *conduction band*.^24^ Some believe that electrons can move between the conduction and valence bands without energy transfer.^22^ Using the METCS concept inventory, Jelovica uncovered that students believe that doping decreases the band gap between the valence and conduction bands rather than increases the number of charge carriers.^25^

### Temperature

Many students also do not understand how the conductivity of metals and semiconductors is affected by temperature.^17–19,24,25^ Students think that metals and semiconductors have the same temperature dependence^18,25^ or that an increase in conductivity with increasing temperature is because electrons move faster at higher temperatures.^17,18,24,25^ Many students find it challenging to describe the temperature-dependent behavior of conductivity.^19^

In contrast to the prior work reviewed, we explored electrical conductivity in the context of a foundation-level inorganic chemistry course where band theory is taught through a molecular level perspective and linked to molecular orbital theory. Students were asked to “describe” how the electronic structure of the two different materials led to the behavior shown in a graph of conductivity versus temperature (see below). Importantly, students were encouraged to explain the connection between the electronic structure of each material and its conductivity as a function of temperature.

## Conductivity in Solids: An Inorganic Chemistry Perspective

Metals and semiconductors can be classified based on differences between the relative magnitudes and temperature dependences of their conductivities (see Figure 1). Metals have higher conductivities than semiconductors. When the temperature increases, the conductivity of metals decreases, while the conductivity of semiconductors increases. These differences in behavior can be explained by band theory.

**Figure 1:**
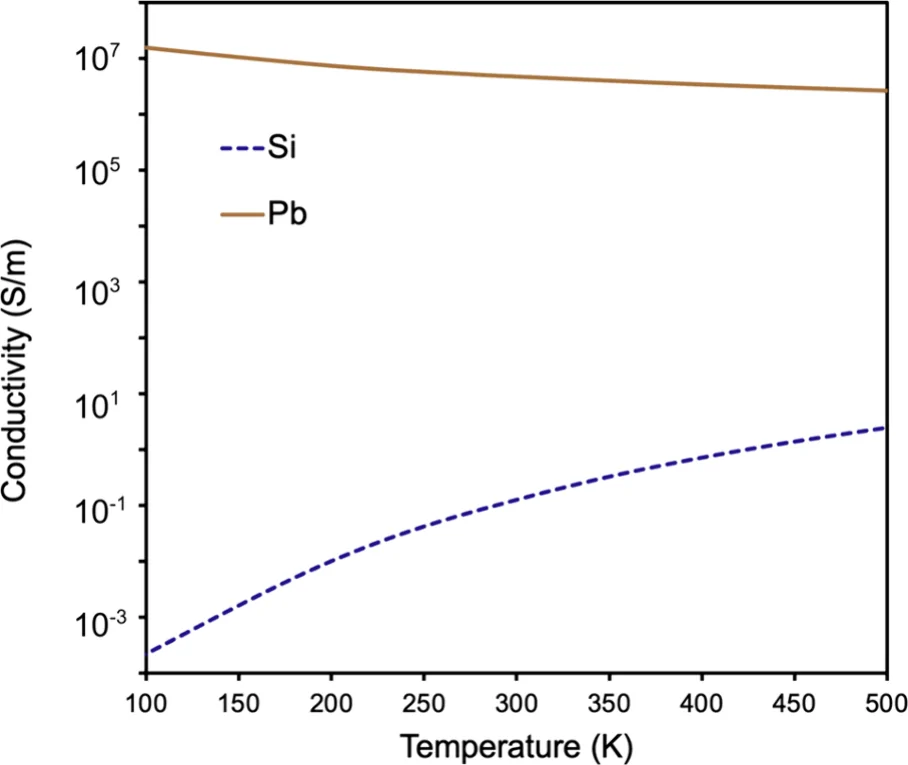
Temperature-dependent conductivity data of silicon (Si) and lead (Pb). Both are group 14 elements with a diamond crystal structure; silicon is a semiconductor while lead is a metal. This figure was constructed using data from the Handbook of Physics and Chemistry and Ramos Cabal.^48,49^

For any material, electrical conductivity involves the movement of electrons (or holes). Electrons in solids do not occupy discrete energy levels as they do in molecules; rather, solids possess a significant number of energy levels that are separated by very small energy differences. These energy levels are called *bands*. Example band energy diagrams for semiconductor and metal materials are shown in Figure 2. Semiconductors like Si, Ge, GaAs, and CdSe are isostructural with the diamond structure, and electrons occupy the lower energy valence band of energies (see Figure 2A). Electrons in this band are separated from the empty conduction band by the band gap energy, *E*_g_. In the band gap, there are no accessible electronic levels. When an electron in a semiconductor is promoted by sufficient energy (i.e., thermal energy, light, or an applied potential) to the conduction band, a “hole” is formed in the valence band (see blue circle in Figure 2A). Metals like Na, Mg, Fe, Cu, and Pb (see Figure 2B) do not have an appreciable band gap. For metals, thermal energy alone is sufficient to excite electrons into an unoccupied energy level (see blue circle in Figure 2B).

**Figure 2:**
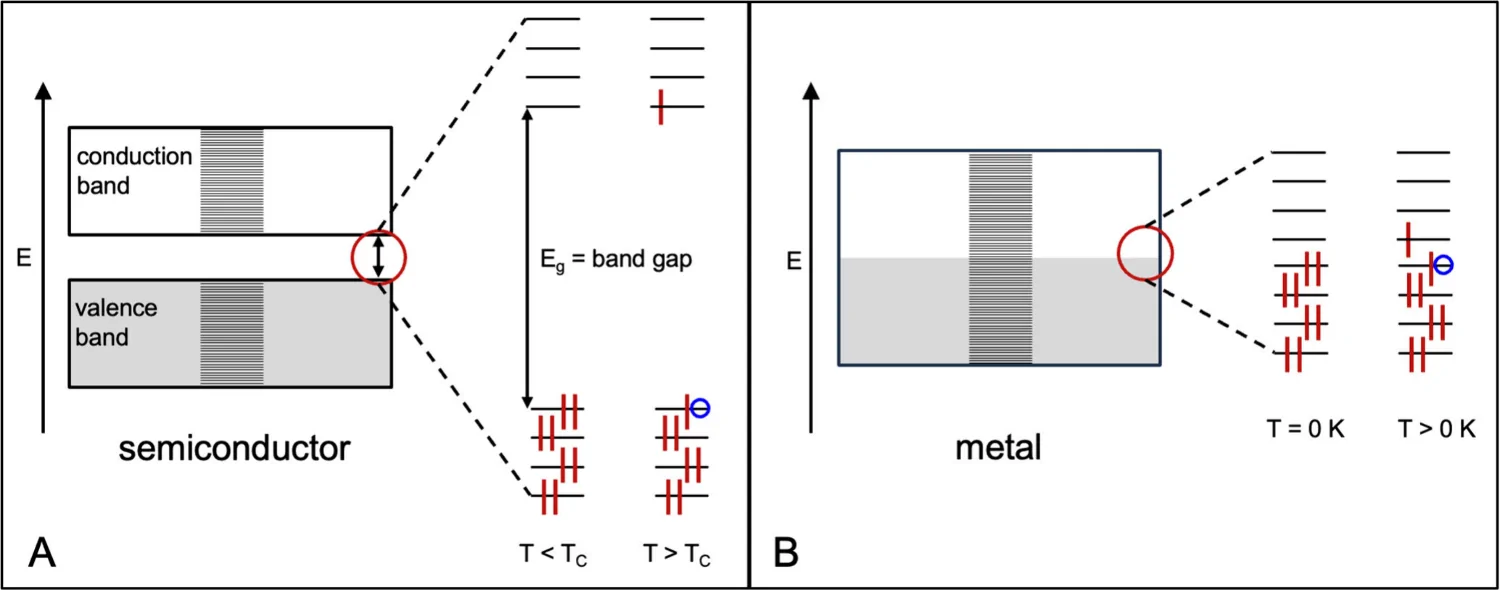
Submicroscopic representation of (A) a semiconductor and (B) a metal. The red lines indicate electrons; the blue circles indicate holes/electronic vacancies, and shading is used to indicate the energy levels that are occupied by electrons in the lowest energy state. *T*_c_ is the minimum temperature at which there is sufficient energy to promote an electron.

The conductivity of a semiconductor increases as a function of temperature because as temperature increases, a greater number of electrons have sufficient energy to be promoted from the filled valence band to the empty conduction band. There are many open and energetically accessible states that the promoted electrons can occupy, which allows for electron flow in the presence of a voltage (i.e., potential energy) difference across the material. In contrast, the conductivity of a metal decreases as a function of temperature because phonons (i.e., lattice vibrations) increasingly impede electron flow, causing the conductivity to decrease. While increasing phonon scattering with increasing temperature also plays a role in reducing the conductivity of semiconductors, the impact is minimal compared to the increase in the number of electrons with sufficient energy to cross the band gap.

The question in this study (see below) involves germanium and Ga-doped germanium. Ga-doped-Ge has a conductivity that is 2 orders of magnitude larger than pure Ge and a conductivity that decreases with increasing temperature, while the conductivity of pure Ge increases with increasing temperature. The data indicate that Ge is a semiconductor and Ga-doped-Ge is a metal.

## Conceptual Framework

Constructivism provides a foundation for research on learning by positioning knowledge not as a fixed body of facts to be transmitted but as meaning that is actively constructed by learners as they interpret experiences through their existing cognitive structures and the practices, language, and representations of the discipline.^50–56^ From a constructivist perspective, learning is a process of sense-making in which individuals integrate new information with prior knowledge, revise misunderstandings, and develop increasingly sophisticated understandings.^57–59^ Constructivism emphasizes how learners build mental models through processes such as assimilation and accommodation, highlighting the importance of developmental readiness.^52,53,59–62^ As students progress in their education, we would expect to see greater integration of ideas into their existing knowledge structures.^54,62,63^

Our findings are interpreted through the lens of the resources framework, which proposes that knowledge consists of many small, context-sensitive elements, i.e., resources.^12–14^ Students activate different cognitive resources to reason through a problem, and different resources are activated depending on the context of the problem. Because these resources often exist as a weakly organized structure, context matters and explains why students reason productively in some situations but inconsistently in others. Through learning, students refine, coordinate, and stabilize their resources into more stable knowledge structures, and our role as instructors is to help students develop a denser and more organized resource base so they can move to a more coordinated knowledge base.

To support student learning, we need to understand the resources that students activate. To that end, we designed an open-ended question that elicits student and faculty member ideas about conductivity in solids and provides information about how students at different levels in the curriculum engage with this material.

## Research Questions

The following research questions are addressed in this study:

- What ideas do inorganic chemistry students and faculty invoke to explain electrical conductivity?
- How does the placement of the inorganic course in the curriculum impact the ideas that are invoked?
- How frequently are submicroscopic models used in student and faculty explanations?

## Research Methods

The work herein was approved by the Human Subjects Review Board (HSRB) at Hope College (HSRB Study 5b199d2f8fb09) and/or by an Institutional Review Board at each of the seven study sites.

### Open-Ended Question Development

The development of the solids-focused assessment prompt (Figure 3) was a collaborative effort, mirroring an open-ended molecular orbital bonding question described previously.^64^ Two inorganic chemistry instructors and one chemistry education researcher crafted an open-ended question to generate a diverse range of free responses from undergraduate students regarding the electronic structure of solids and conductivity. The initial question was pilot-tested in spring 2018 with 57 students enrolled in inorganic chemistry courses at four different four-year degree-granting institutions. Results demonstrated that the question effectively elicited student responses that addressed the intended concepts of solids, electronic structure, and conductivity. While the quality of student responses varied, the question was not revised, as it successfully generated meaningful data.

**Figure 3:**
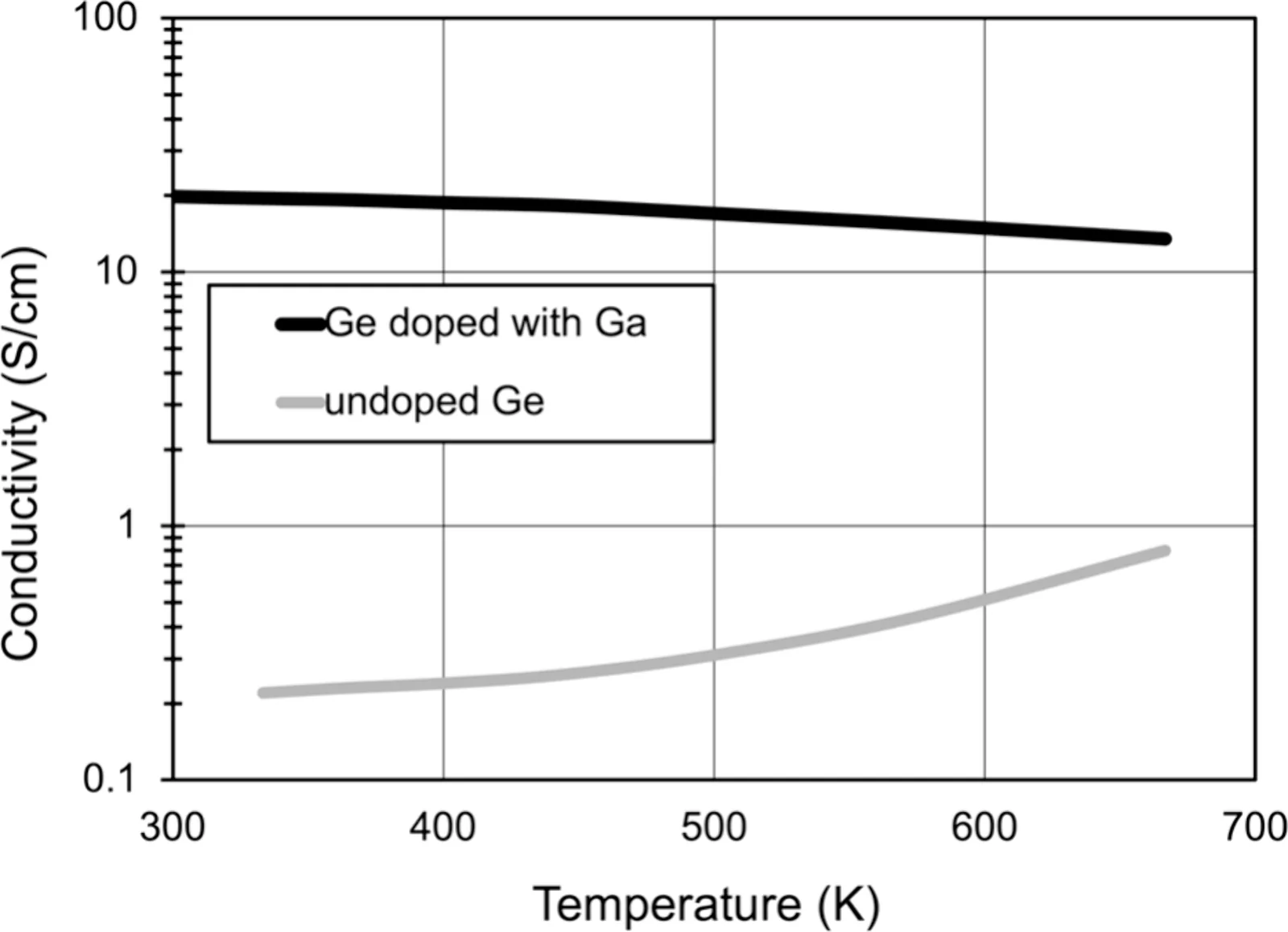
Conductivity data provided to students for the open-ended solids question. This figure was constructed using data from Stoib et al.^65^

The open-ended question provided students with a graph based on data from the primary literature (see Figure 3)^65^ and asked them to respond to the following prompt: *“Below is a graph of the conductivity as a function of temperature for undoped Ge and Ge highly doped with Ga. Using complete sentences and drawing any additional diagrams or graphs you need, describe how the electronic structure of these two solids leads to differences in the observed conductivity.”*

### Classroom Use of the Open-Ended Solids Question and Data Collection

The open-ended question was provided to seven inorganic chemistry instructors from multiple institutions within the United States, including four-year degree-granting institutions through graduate-degree-granting institutions. The instructors who participated in this study were among the 63% of inorganic chemistry instructors who report covering band theory in their foundation courses.^7^ In all contexts, individual instructors administered the question to students enrolled in their foundation-level inorganic chemistry course.

Instructors were told to have their students answer the open-ended solids-focused question in class during their solids unit at any time *after* they had covered the electronic structure of solids. The specific details regarding question administration were left to the instructor, allowing them to implement it meaningfully within their instructional context and goals. Instructors were also advised to have students complete the question *individually* as an in-class activity. The research team suggested several ways the question could be used during class (e.g., as part of a think-pair-share activity, an individual activity followed by a whole class discussion, a quiz); ultimately, the decision on how to administer the question was left to the instructor. After implementation, instructors scanned students’ responses without identifying information and sent these to the research team for analysis. In all cases, student data resulted from individual, initial responses to the question; any revisions or discussions after the collection of individual responses were not captured, ensuring the data represented individual student ideas. Lastly, instructors were asked to answer the open-ended question themselves as a method for generating ideal student responses and provide context for their own students’ explanations.

### Data Analysis

Data were inductively analyzed through an iterative process involving multiple coders to facilitate consistency and clarity in the code development and application.^66–68^ The initial codebook focused on the effect of temperature on conductivity and was initially developed by two raters using 10% of the data set. After initial code development and agreement between the two raters, a third rater tested the codebook by applying it to a second subset of the data (20% of the total). Through discussion among the three raters, code descriptions and applications were revised. This iterative approach of revising the codebook and testing it on a subset of the data set continued through multiple rounds, with up to five different raters, until 100% agreement was reached on all code applications across the entire data set; regardless of the number of raters, all code applications were conducted by a minimum of two raters with all disagreements resolved through group discussion until 100% agreement was reached. This iterative approach was repeated for a second analysis/codebook focused on conductivity and electronic structure; this process employed four different raters with all code applications conducted by at least two raters. All disagreements were resolved through whole-group discussion and consensus. The two analyses were then merged to consider overarching categories across responses, providing deeper insights into how students considered the electronic structure of solids in the context of the conductivity and temperature data (Table 1).

**Table 1:** Ideas Discussed by Respondents with Corresponding Example Student Quotes^a^

Category: Overarching Concept	Subcategory: Specific Idea Discussed and Coded	Representative Student Quote
Conductivity (C)	C.0 No discussion of conductivity.	“GaGe is an n-type semiconductor making the energy going from VB to CB a lot easier and more efficent [*sic*] that without doping Ge.” *Student 7.125*
	C.1 Discussion of any electron motion at all: *electrons jump or move between bands, orbitals, or atoms; being promoted; anything related to electron motion*.	“...it’s harder for the electrons to move, so conductivity decreases...” *Student 7.104*
	C.2 Makes a connection between conductivity and temperature: *terms may be vague or respondents might say “heated”.*	“...as the temperature rises, the conductivity slowly decreases...” *Student 6.003*
	C.3 Discussion of any hole motion: *related to holes in bands*.	“The holes are able to hold a current...” *Student 5.010*
Doping (D)	D.0 No discussion of doping.	“High conductivity in anode materials is necessary for transfer of electrons & Gallium greatly reduces thermal conductivity” *Student 7.135*
	D.1 One or both of the materials is/are classified, named, labeled: *a p- or n-type semiconductor, conductor, metal, semiconductor - classification must be in the context of doping*.	“It seems that undoped Ge is a semi-conductor and the Ge doped with Ga acts like a metal/conductor...” *Student 4.010*
	D.2 Discussion of the fact that conductivity changes with doping: *discussion of conductivity differences before/after doping, not just one or the other*.	“Ge doped w/ Ga has a much higher conductivity...” *Student 2.005*
	D.3 Mentions that a new band is created by doping: *they can describe the band in words or represent it with a picture (has to be associated with Ga-doped Ge)*.	“The doped Ge has an extra band from the trace amounts of Ga....” *Student 1.102*
	D.4 Doping changes the number of charge carriers (electrons or holes).	“Since Ga has one less electron than Ge, it adds holes in the valence band...” *Student 3.003*
Band Structure (BS)	BS.0 No discussion of band structure. *If this code is chosen, the response can, at most, have BS.5 as well*.	“Ge undoped has more electrons than if you were to replace some Ge with Ga. Thus GeGa is a p-type conductor.” *Student 7.117*
	BS.1 Discussion of the existence of a band gap but nothing beyond that. *If this code is chosen, the response can, at most, have BS.5 as well*.	“Ge doped with Gallium has a band gap that is compatible with UV. This increases the structure’s absorption ability, thus increasing conductivity.” *Student 7.109’*
	BS.2 Discussion of charge carriers in relationship to the band.	“...meaning that the band gap has been decreased resulting in an easier flow of electrons...” *Student 6.007*
	BS.3 Discussion of changes in the energy gap, such as size of elements affecting band gap size, effect of temperature on band gap, effect of extra electrons on band gap. *This code is not applied if the student only said a new band is created, that is code D3*.	“...there is an effective reduction in the band gap/E needed to conduct electricity...” *Student 5.003*
	BS.4 Uses the language of molecules such as orbitals, HOMO/LUMO, references to s and p orbitals.	“...the Ga impurities decrease the energy level between the s-orbitals and p-orbitals...” *Student 1.001*
	BS.5 Any picture/sketch that shows something about bands: *the drawing must be clearly associated with bands*.	
Temperature (T)	T.0 No discussion of temperature.	“Gallium is a p-type dopant for Germanium enusing [*sic*] holes in the valence band resulting in higher conductivity.” *Student 3.001*
	T.1 General statement that connects temperature and conductivity data.	“The temperature on the graph indicates that Ge by itself would be more conductive on its own...” *Student 3.005*
	T.2 Discussion of how increased temperature promotes electrons to higher energy levels or across the band gap.	“...As temp. increases the undoped Ge is able to excite more e- to the conduction band increasing conductivity...” *Student 3.004*
	T.3 Discussion of how or why increased temperature leads to a decrease in conductivity for metals: i.e., via dissipation of electron energy through phonon vibrations.	“The doped Ga declines slightly with heat because the heat is causing vibrations disturb the flow of electrons...” *Student 1.102*

a Student quotes with ellipses are excerpted from the full responses. Full student responses are included in Table S2.

While measures of interrater reliability are common in qualitative studies, the author team chose to focus on 100% agreement, as opposed to a calculated statistic. Four or five raters, depending on the analysis, considered different perspectives and came to consensus by resolving all disagreements, adding greater trustworthiness to the interpretations and conclusions.^69,70^ Reaching 100% agreement with four or five raters is a difficult process but resulted in more nuanced codes, code descriptions, and trustworthiness than using a calculated coefficient for two raters. Detailed documentation of this method was maintained throughout data analysis, ensuring that the team understood the decisions made, could track when and how disagreements were resolved, and could reflect on the overall interactive process. This level of transparency adds credibility to the findings. Lastly, because the data are reflective of 117 students and seven instructors from seven different institutional contexts, the patterns found in the data are more credible than if data were collected from a single classroom. Our focus on embedding credibility, dependability, and confirmability within the study methods helps support the overall trustworthiness of the findings and adds to the transferability of findings to other contexts.

As the course contexts primarily differed by whether they required a physical chemistry prerequisite, Fisher exact tests were employed to compare the prevalence of ideas by this prerequisite grouping variable. Given the exploratory nature of this project and the 19 simultaneous comparisons, the Benjamini-Hochberg (B-H) Procedure was employed to reduce the false discovery rate (i.e., minimize Type I error).^71,72^ This approach is preferable to a Bonferroni correction given the exploratory nature of the project and concerns about false negatives (i.e., Type II error).

For context, the B-H Procedure ranks raw *p*-values in ascending order and then calculates a B-H critical value by dividing each *p*-value’s rank by the number of tests and multiplying by an acceptable false discovery rate; in this context, we chose a false discovery rate of 5%. If the raw *p*-value is less than the B-H critical value, it is considered significant–including all other *p*-values ranked higher. A full table of *p*-values and B-H critical values for all comparisons is provided in Table S2; only the top five comparisons were significant according to the B-H procedure. As an additional method to understand the context of student responses, videos of class sessions and course artifacts (e.g., PowerPoint slides, in-class materials, etc.) provided by the instructors were examined. These confirmed that all students had course experiences that addressed the four overarching categories of ideas.

## Results and Discussion

### Ideas Invoked by Respondents

From our iterative analyses of student and instructor responses, four overarching categories were identified based on topic areas discussed: conductivity (C), doping (D), band structure (BS), and temperature (T) effects. It is interesting to note that these ideas match the enduring understandings from the Anchoring Concepts Content Map (ACCM) for inorganic chemistry, a consensus model developed through focus groups with representative faculty members.^72^ Additional details about the ACCM enduring understandings can be found in the Supporting Information.

Each category of ideas was further subdivided into specific ideas that characterize the ways respondents addressed the open-ended question and data. Table 1 presents the four categories and 15 ideas identified across the data corpus, along with excerpts from student responses that demonstrate the ideas; an individual respondent may or may not have addressed all four categories in their written responses. A complete description of each idea and full quotes of example responses are included in Table S2.

The number of categories incorporated into a response indicates the kinds of resources that are being activated, i.e., the concepts that students and faculty find germane to a discussion of the data, while the subcategories describe how specific ideas are applied to the problem. Figure 4 shows the number of categories addressed in student and instructor responses. For all analyses, codes C.0, D.0, BS.0, and T.0 were omitted, since they correspond to responses that did not mention any ideas in their respective category. Idea BS.5, sketching a picture, was not included in these totals, because it is not a part of the text-based explanation. Overall, results show that instructors find all concepts (i.e., conductivity, doping, band structure, and temperature) relevant when responding. While students tend to address fewer categories than instructors, every student addressed at least one of the coded categories in their response.

**Figure 4:**
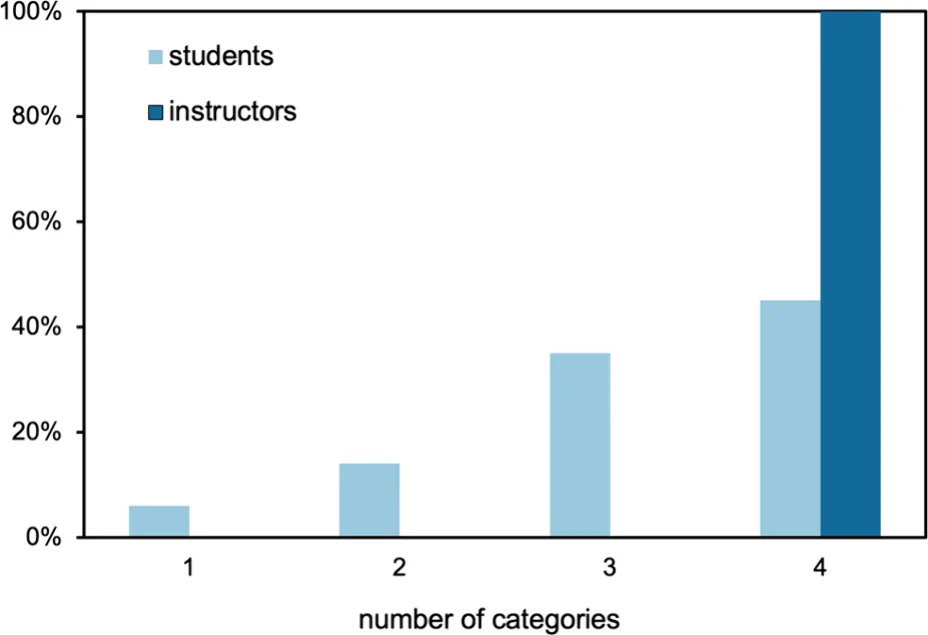
Percentage of all students (*N* = 117) and instructors (*N* = 7) who discussed one or more of the four categories: conductivity, doping, band structure, and temperature.

Figure 5 shows the percentage of students that incorporated each of the four categories into their response. Nearly all of the students (99%) discussed doping. Most students addressed conductivity (88%), while fewer discussed the band structure (74%) and temperature (61%). It is not surprising that most students discussed doping and conductivity, as these were explicitly mentioned in the prompt. It is surprising that nearly 40% of students did not discuss temperature since it was also included in the prompt, and the graph showed the temperature dependence of conductivity. Although the band structure was not mentioned in the prompt, it is encouraging that three-quarters of the students made the connection to band structure, the underlying submicroscopic explanation for these observations.

**Figure 5:**
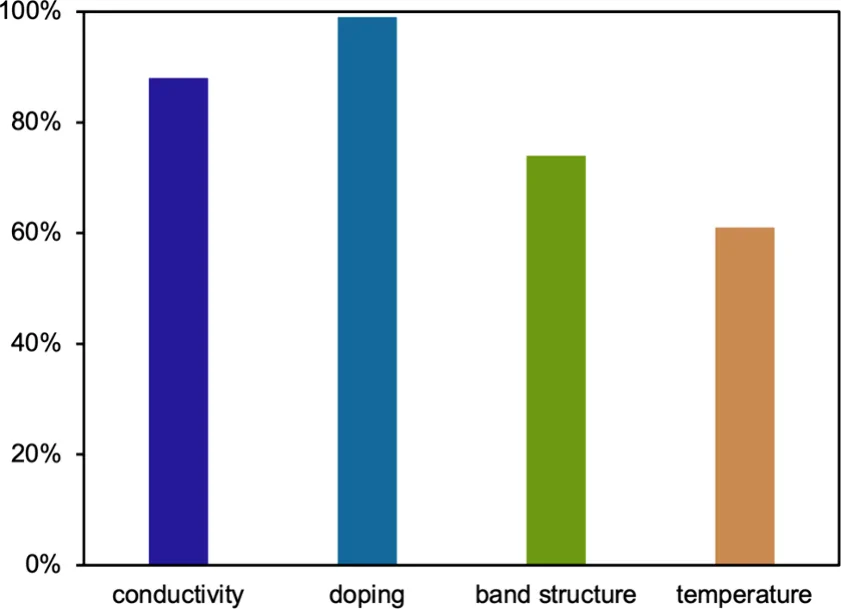
Percentage of all students (*N* = 117) who incorporated each category–conductivity, doping, band structure, and temperature–in their response. All instructor explanations included ideas from all categories.

### Domain Knowledge

Domain knowledge is important because it shapes how students learn, use, and interpret new information; researchers have linked high prior knowledge to an ability to focus on conceptually relevant features and connect information and representations.^73^ While activating more ideas does not guarantee the presence of a more integrated knowledge structure, without prior knowledge, people cannot develop integrated knowledge structures. The inclusion of more ideas can serve as a proxy for a greater knowledge of the material.

The subcategories represent the specific ideas that participants include in their responses, and many of these require integration and application of concepts. The number of ideas, as shown in Figure 6, provides insights into respondents’ domain knowledge. Four possible idea codes contributed to the number of ideas in the doping category, while all other categories had three possible idea codes. In their responses, students discussed the greatest number of ideas about doping (avg. = 2.1 out of 4), followed by conductivity (avg. = 1.4 out of 3), band structure (avg. = 1.1 out of 3), and temperature (avg. = 0.9 out of 3).

**Figure 6:**
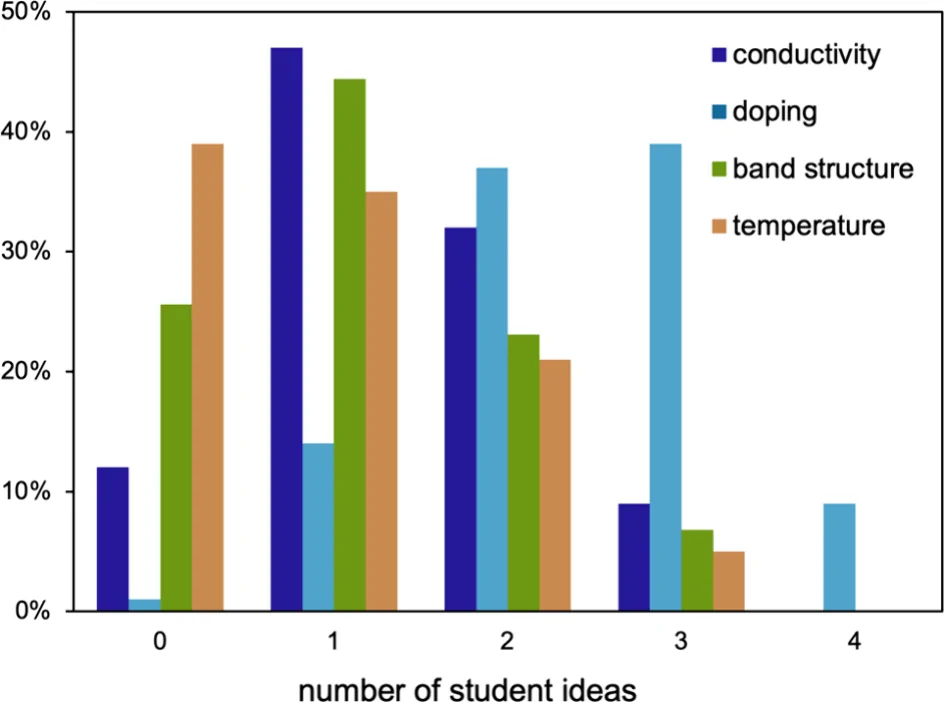
Percentage of all students (*N* = 117) who discussed 0, 1, 2, 3, or 4 ideas related to conductivity, doping, band structure, and temperature. There was a maximum of four possible ideas related to doping and three related to the other categories.

#### Impact of Curricular Placement

There are differences in the number of ideas included in a response when curricular placement is considered (see Figure 7). The placement of the foundation-level inorganic course in the chemistry major curriculum varied at the seven institutions in the study. Institutions were categorized into groups based on if the inorganic course required students to take a physical chemistry course (either quantum or thermodynamics) as a prerequisite (“p-chem prereq”, 4 institutions) or those that did not require physical chemistry prior to enrollment in the inorganic course (“no p-chem prereq”, 3 institutions). It is important to note that these groups were determined using official prerequisites; it is possible that students in the “no p-chem prereq” group may have taken physical chemistry previously, while students in the “p-chem prereq” group may not have taken at least one semester of physical chemistry. Figure 7 shows that instructors included a greater number of ideas than students and that students in courses that require a physical chemistry prerequisite included more ideas in their responses than students enrolled in courses where they were not required to have taken physical chemistry. Of the 13 ideas that were coded in the data set, on average instructors included 10.6 ideas, students in a course with a physical chemistry prerequisite included 6.5 ideas, and students in a course without the physical chemistry prerequisite included 5.2 ideas. While many of these differences are not significant, there is a general trend between a greater number of ideas included in responses and more experience in chemistry.

**Figure 7:**
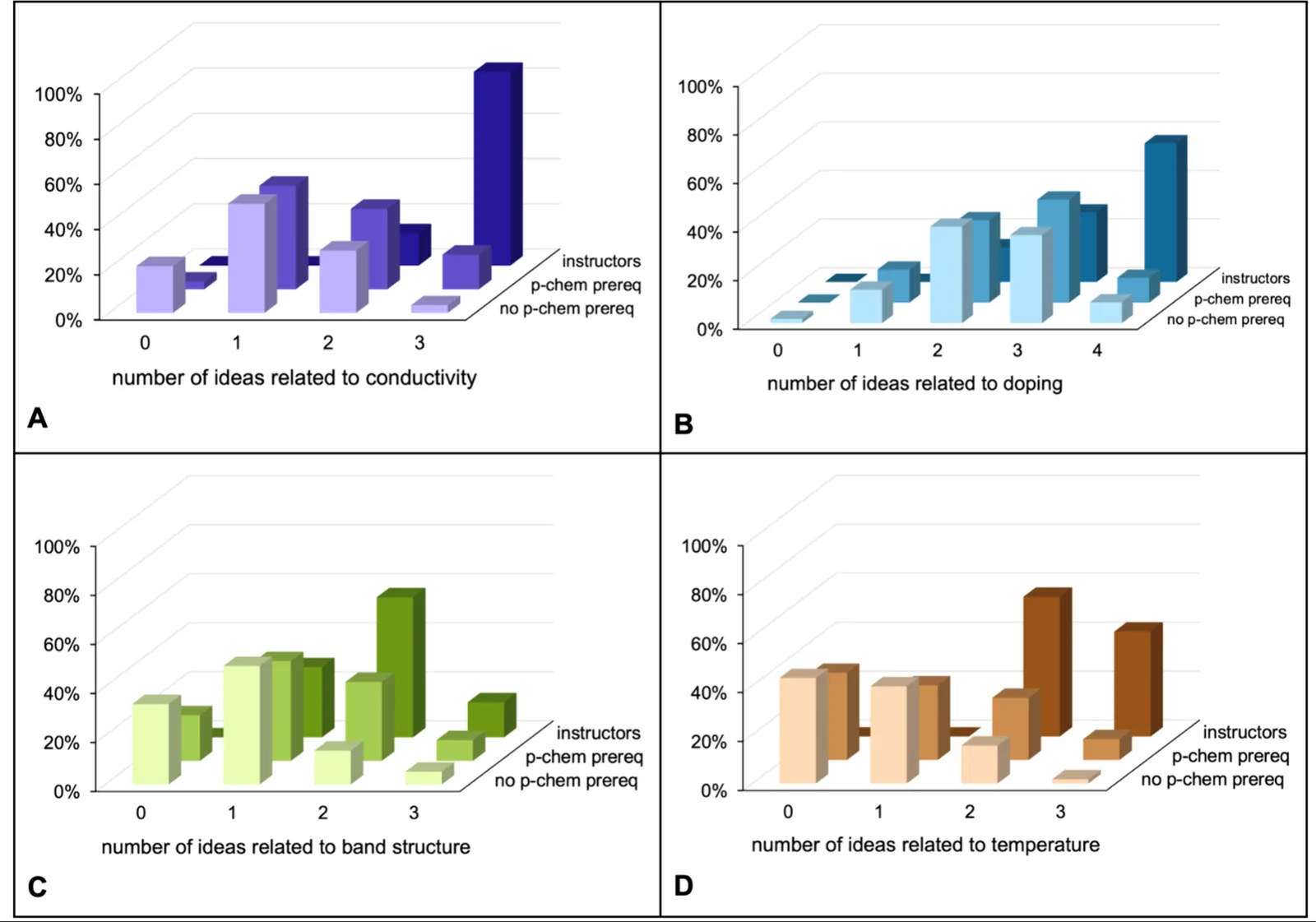
Percentage of students enrolled in courses without a physical chemistry prerequisite (*N* = 58), students enrolled in courses with a physical chemistry prerequisite (*N* = 59), and instructors (*N* = 7) who discussed 0, 1, 2, 3, or 4 ideas related to (A) conductivity), (B) doping, (C) band structure, and (D) temperature.

Developing expertise changes what people notice and how they acquire, organize, represent, and interpret information. Experts notice more features and meaningful patterns of information, have more coordinated knowledge, can more flexibly retrieve this knowledge, and make more useful connections in their knowledge.^74^ The more content-rich responses of instructors illustrate their greater knowledge base and their ability to extract information from the data provided.

We attribute the differences among students to their progression in the chemistry major. As students progress through the chemistry degree, they acquire more information that becomes available to them to address problems. They also gain more experience using representations as tools for explaining chemical phenomena. Students enrolled in inorganic courses with a physical chemistry prerequisite have likely taken more chemistry courses. Perhaps these students and instructors provided more complete responses to this open-ended question because of their greater experience studying chemistry and interpreting data.

#### Inclusion of Submicroscopic Ideas

Figure 8 presents the percentage of instructors and students, in courses with and without a physical chemistry prerequisite, that included the specific ideas in Table 1 in their responses. The small number of instructors (*N* = 7) limits our ability to make claims about significant differences between instructors and each group of students.

**Figure 8:**
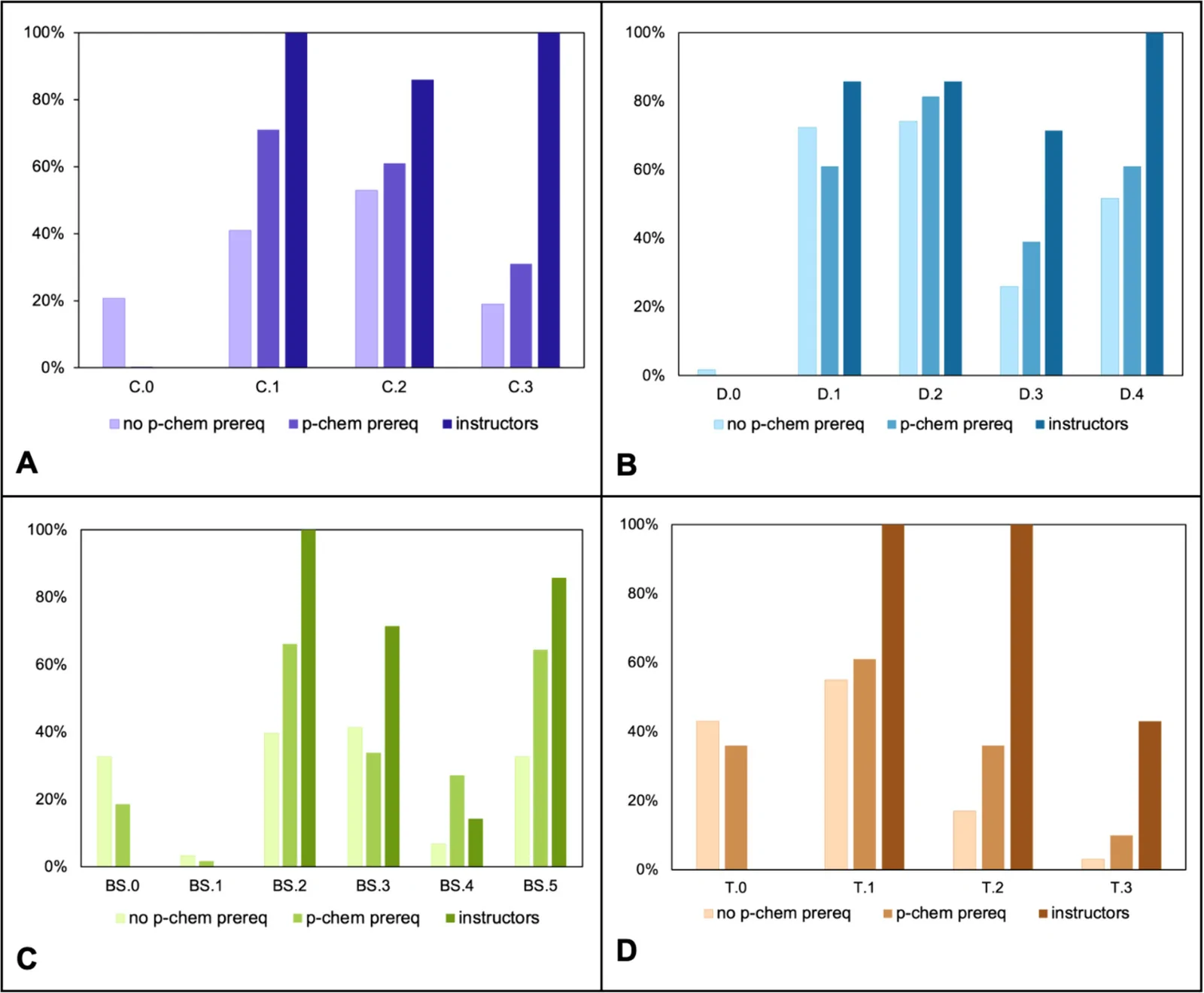
Percentage of students enrolled in courses without a physical chemistry prerequisite (*N* = 58), students enrolled in courses with a physical chemistry prerequisite (*N* = 59), and instructors (*N* = 7) who mentioned any of the ideas related to (A) conductivity, (B) doping, (C) band structure, and (D) temperature in their response to the open-ended question. Totals do not add to 100% across a given category as many students and instructors addressed more than one idea in their response. The ideas are detailed in Table 1.

One observation when comparing responses is that instructors are more likely than students to employ submicroscopic models. Several ideas coded were directly related to submicroscopic phenomena, including the discussion of electron and hole motion (ideas C.1 and C.3), charge carriers (ideas D.4 and BS.2), and the mechanism of conductivity (ideas T.2 and T.3). All instructors discussed five of these six ideas, with the exception being idea T.3, the explanation for why metal conductivity decreases as a function of increasing temperature.

Aside from idea D.1, *classification of compounds*, and idea BS.3, *impact on band gap*, students in a course with a physical chemistry prerequisite incorporated more ideas in their response than students in a course without this prerequisite. This pattern generally holds across the data corpus with five ideas statistically different according to the B-H Procedure: ideas C.0, C.1, BS.2, BS.4, and BS.5.

These results suggest that students in courses with the physical chemistry prerequisite discussed conductivity (idea C.0) and electron motion (idea C.1) significantly more frequently than students in courses without the prerequisite, and students in courses with the physical chemistry prerequisite were more likely to draw a picture while explaining the band structure of the materials (idea BS.5). More advanced students were also more likely to discuss charge carriers in relation to the band structure (idea BS.2) and use the language of molecules when describing the conductivity of the two materials (idea BS.4). It is notable that all of these ideas, including incorrect idea BS.4, invoke submicroscopic phenomena. Like instructors, students who have had physical chemistry are likely further along in their course of study and are more likely to have greater chemical knowledge. Additionally, these students were more likely to use a representation in their responses (see below).

While all instructors discussed the differences in conductivity and temperature of the two materials, few students discussed both differences, and some students addressed neither. Students without the physical chemistry prerequisite were less likely to discuss conductivity (idea C.0), which is a central concept in this problem. Conductivity and temperature are the key physical properties that allow the electronic behavior of these materials to be distinguished, and the absence of conductivity discussions by students without physical chemistry suggests that they may not recognize key features in the problem.^74^

While differences observed between the groups of students may be related to prior exposure to content in the physical chemistry prerequisite course, we believe this is unlikely because of the molecular focus of the chemistry curriculum and the small number of physical chemistry courses that address solids.^27^

The ability to connect a macroscopic property with submicroscopic explanations is an indicator of having a more coherent, integrated knowledge structure.^75–77^ An expert-level response should invoke the submicroscopic ideas of band theory. We interpret the increase in the inclusion of submicroscopic explanations as students progress in the curriculum and between students and instructors as indicative of more time studying and conducting chemistry. This pattern mirrors expert-novice differences commonly seen between instructors and students.^74^

### Use of Visual Representations

Chemistry is a field rich in the use of models and visual representations to communicate ideas and concepts, and the open-ended question employed in this study explicitly prompted respondents to draw representations to support their answers. While learning chemistry, students may be asked to produce a drawing to assess their knowledge of a specific chemical representation. However, scientists use representations as a means to an explanation or prediction, and Stowe et al. encourage us to ask students to do the same.^78^ The prompt, in this case, did just that, asking respondents to include any representations they “need” to describe how electronic structure leads to differences in conductivity. In this context, in addition to examining written responses to the open-ended question, we also analyzed the visual representations generated to accompany answers.

Visual representations were generated by 6 of the 7 instructors (86%) and by 63 out of 117 students (54%). Overall, band diagrams were the most prevalent representation drawn by respondents to help communicate and explain their ideas (see Figure S1). Of the 6 instructors who included pictures, all drew one or more band diagrams. Of the students who drew a picture (idea BS.5), 90% drew a band diagram; most of the remaining students drew Lewis structures (6%). There was great variation in the way students represented band diagrams, but this is not surprising, since there are multiple variations of the visual representations used to depict band diagrams.

Students with the physical chemistry prerequisite drew a picture more frequently when explaining the band structure of the materials (idea BS.5) compared to students without the prerequisite course. The increased use of pictures/drawings in responses by these students and by the instructors may arise from an enhanced ability to use representations to describe the submicroscopic behavior of systems, which has been observed as chemists become more expert-like.^54,79^ Overall, the presence of visual representations is encouraging, as it provides additional evidence that respondents are trying to provide submicroscopic explanations for conductivity. These representations may also indicate an attempt to organize their knowledge.

Since respondents were encouraged but not required to draw in support of their answers, it is challenging to conduct detailed analyses of the features of the representations or make overarching claims. However, on average, regardless of course prerequisites, students who included a representation included 2.5 submicroscopic ideas in their response compared to the 1.9 submicroscopic ideas of students who did not include representations and were more than twice as likely to discuss electron motion. More work is needed that purposefully investigates how faculty and students generate and use representations of the electronic structure of solids and how they can help students understand the role that electron motion plays in conductivity.

### Content-Based Ideas

While our final coding scheme focused on categorizing ideas rather than the accuracy of ideas, an earlier coding scheme categorized the accuracy of responses. While this coding scheme was discarded because multiple responses required inferences during coding, we read individual responses deeply and noticed several important, overarching themes.

#### Classification

We noticed that instructors classified materials more frequently than students by assigning them a label (such as metal, semiconductor, etc.) and classified them in a scientifically accepted way. While all instructors classified both materials, students classified the materials only half of the time. Classifying elements in a problem is often a productive first step in problem solving. We believe the difference in the number of respondents who classified the materials is because instructors more frequently characterized the material as they have the problem-solving experience to know that characterization helps them construct an effective explanation. The experts (i.e., instructors) are better able to notice features and meaningful patterns of explanations that are not always invoked by the novices (i.e., students).^74^

All instructors classified pure Ge in a way that is at least partially consistent with the data; however, fewer than half of students classified pure Ge as a semiconductor. Instructors more frequently recognized that Ge is a semiconductor because of their knowledge of descriptive chemistry or knew that the change in conductivity as a function of temperature is consistent with Ge being a semiconductor. However, only three of seven instructors, and fewer than one-quarter of students, classified Ga-doped Ge correctly as a metal or conductor; most classified it as a semiconductor or mentioned that it was doped. In many textbooks, Ga-doped Ge is presented as a p-doped semiconductor. A novice could associate the phrase “Ga-doped Ge” with a p-doped semiconductor. However, this is only true for the *lightly*-doped material. The data presented in the open-ended problem, which is for a *heavily*-doped material, shows metallic or conductive behavior.

On the basis of the number of responses that were inconsistent with the conductivity data provided, we infer that some respondents focused on the surface features of the problem (i.e., the words), rather than incorporating the data into their response. Research shows that experts interpret figures and representations more deeply, while novices tend to focus on surface features such as the words in the prompt.^80,81^ The words used may cue ideas about the materials that are incongruent with the conductivity data.

#### Mechanism of Conductivity

There were differences between how respondents discussed the temperature-dependent behavior of pure Ge compared with Ga-doped Ge. While all instructors correctly used the band theory model to describe the temperature-dependent conductivity of the semiconductor Ge, only the three instructors who correctly characterized the Ga-doped Ge material as a metal or conductor used band theory to correctly explain the temperature-dependent behavior. The other instructors did not link their response to band theory or provided a response that was consistent with their classification of the material as a semiconductor.

Both students and instructors struggled to discuss the electronic properties of Ga-doped Ge. We did not see this difficulty in instructor responses when instructors discussed Ge or when instructors responded to another open-ended question on bonding.^64^ This observation suggests that their knowledge of the electronic structure is activated in a different context (a lightly doped system), revealing that respondents (including instructors) could lack deep knowledge, which impacts their ability to correctly classify and describe the doped material with band theory. This is also supported by our survey data, where instructors report not being comfortable with solid-state chemistry.^9^

#### Idiosyncratic Ideas

While there were many correct ideas in the analyzed responses, we noticed several idiosyncratic ideas about the electronic structures of the solids. While the fixed nature of data collection did not allow us to determine whether all previously identified misconceptions were held by students, responses suggest that students had trouble connecting macroscopic behavior and submicroscopic explanations, similar to the engineering and physics education literature.^16–18,20–22,24^ Students had difficulty expressing how conductivity is affected by temperature^17–19,24,25^ and how doping affects conductivity.^21,24^ This indicates that they may lack the content knowledge or resources to attend to these features. Unlike physics and engineering students,^20,21^ many inorganic chemistry students used the band-structure model in their responses. This difference may arise from the fact that most chemistry students do not work extensively with the Drude model prior to learning the band structure model and do not have a prior naive model to fall back on. Examples of scientifically incorrect ideas from inorganic chemistry students are reported in Table S3.

## Limitations

The question used in our study (see Figure 1) was an open-ended prompt asking students to *“describe how the electronic structure of these two solids leads to differences in the observed conductivity”* in response to a graph demonstrating conductivity vs temperature behavior for two materials. Respondents were not provided with any scaffolding to direct their responses, resulting in the observed variable use of the graph/data provided to support their answers. As such, we were only able to evaluate the ideas respondents shared related to the electronic structure and conductivity of the materials. Additionally, the open-ended nature of the question and the mechanism for data collection limited the depth of our analysis as we were unable to follow up with respondents, ask clarifying questions, etc. Future work should consider using interview-based approaches which would allow for a more in-depth discussion of student and instructor understanding of these topics and understand the integration and coherence of ideas.

While the findings are limited, it is important to note that we analyzed responses across seven class/institutional contexts, including responses from seven instructors and 117 students at different stages of their chemistry degree programs at different types of institutions. Each class of students took prerequisite courses before enrolling in their foundation-level inorganic course; however, each institution varied in the depth and breadth of topics covered in prior coursework. Data regarding individual class contexts were limited to officially stated prerequisite coursework; as such, we were unable to fully relate in-class experiences to our findings. However, the variety of institutional contexts coupled with the sample size of students suggests that results and salient patterns across responses have more credibility and transferability than if only one institutional context was studied.

## Conclusions and Implications

Four categories of ideas were key to explaining the temperature-dependent conductivity of the solids in the open-ended question used in this work: conductivity, doping, band structure, and temperature. An analysis of responses showed that most students discussed a subset of the four categories present in the question but did not address all four elements. Students more frequently discussed ideas about doping and conductivity than ideas related to the band structure and temperature. In contrast, instructors incorporated all four categories into their responses. Instructors were also more likely to both classify and correctly classify the two solids described in the question. We also found a difference in the domain knowledge and inclusion of submicroscopic ideas in responses, which we interpret as respondents having a greater experience with the material. Instructors invoked the most ideas in their responses, while students invoked fewer ideas. Furthermore, instructors more frequently provided submicroscopic explanations for conductivity and incorporated visual representations in their responses. Differences were also observed between students enrolled in inorganic chemistry courses requiring a physical chemistry prerequisite and those enrolled in courses that did not. Students without a physical chemistry prerequisite less frequently classified materials, included submicroscopic ideas, and drew visual representations of the band structure of solids.

These observations are consistent with differences in the available resources that instructors and more advanced students have compared to those of less advanced students. Instructors and more advanced students more frequently classify material correctly, recognize key problem features, and make connections between macroscopic data and submicroscopic explanations. Furthermore, if the physical chemistry course is taken as a proxy for student progression through the chemistry curriculum, then these observations suggest that curricular sequencing may influence the development of student understanding.

Using the consensus model of pedagogical content knowledge,^82,83^ we provide recommendations for instructors. First, instructors should identify the learning goals for their course and focus on these ideas. Like inorganic chemistry itself, solid state chemistry addresses a broad range of topics. The concepts and level of sophistication used will depend on the placement of the foundation-level inorganic chemistry course. Instructors might consider answering the question “What do I want my students to know and be able to do?” and align their instruction with those goals. Because electronic properties are critical for understanding many modern materials, we believe that, at a minimum, all chemistry students should be able to connect the conductivity of solids with their underlying electronic structure. Specifically, students should be able to (1) describe how bands arise in solids; (2) recognize how band occupancy impacts conductivity; (3) describe the mechanism of conductivity using the band model; (4) predict the electronic properties of elements and simple compounds based on their electron configuration; and (5) predict how dopants will impact band occupancy.

Second, we urge instructors to make connections to the molecular orbital theory that is covered in a foundation-level course. Band theory is an extension of molecular orbital theory applied to a very large number of atoms. Connecting these ideas helps students build a coherent, integrated framework that reduces cognitive load and reinforces the coherence of chemical models. Chapter 7 of *Teaching General Chemistry: A Materials Science Companion* ^84^ provides a readable and operationalizable way to help students relate these ideas by beginning with a diatomic molecule and walking through how the addition of atoms changes the number and energy spacing of the energy levels. Additional activities that make these connections can be found at the Virtual Inorganic Pedagogical Inoranic Resource, VIPEr.^100^

Third, we believe that instructors should use representations that help students visualize the components of a system that are useful for productive explanation and prediction.^78^ In the context of this question, the visual representation of a band structure is central to describing the conductivity of semiconductors and metals. We generated the representation in Figure 3 to connect bands and their underlying crystal orbitals of metals, insulators, and intrinsic semiconductors. This representation encodes the continuum and occupancy of electronic states that can be connected to the impact the electron configuration has on band occupancy, the impact of empty states on conductivity, and the impact of temperature on conductivity. Instructors should help students unpack how electronic states are encoded in the diagram, the meaning of the band gap, and the role that energy plays in the population of the states. Instructors should also help students use representations and the models behind them consistently and correctly. Deliberately drawing attention to the specific features of these representations and the explanations associated with this model provide an opportunity to develop a deeper understanding of the differences between metals, semiconductors, and insulators.

Fourth, we believe that it is essential to help students make connections between macroscopic data and submicroscopic explanations. We believe that having students analyze and connect data to submicroscopic models help them develop a more coherent chemical framework. We recommend showing students conductivity data for a semiconductor and metal (Figure 2) and helping them to identify the features that identify the electronic behavior of the solid: the magnitude of the conductivity and the temperature dependence of the material. We also advocate that instructors use examples directly from the primary literature.^85,86^ Several activities that include data from the primary literature can be found on VIPEr.^87,88^ Instructors can model how classification, which is often a productive first step, can be used to help solve the problem. By engaging with temperature-dependent conductivity data, students can identify differences in conductivity that will allow them to classify the materials. Instructors can help students develop representational competence by incorporating band diagrams into their responses to show how the band structure model can explain the data provided. After instruction, the open-ended problem in this study can be used as a formative assessment to understand whether students are productively interpreting data and using submicroscopic explanations to explain the electronic behavior of the materials.

Finally, we believe that integrating knowledge about extended systems into existing knowledge about molecular systems may help students develop a stronger overall understanding of chemistry. We encourage chemical educators to incorporate extended systems in instruction. While inorganic chemistry courses have historically included a unit on solids, much of the chemistry curriculum is focused on small molecules or intermediate-sized macromolecules. The inorganic course may be the only course in which students explore extended systems. While instructors may be concerned that they do not have the expertise to teach this topic, topic-specific pedagogical content knowledge can be developed.^23^ Solids play an important role in industry and consumer applications, and we believe that their inclusion in the curriculum will help to engage students.

## Supplementary Material







## Notes

Conceptual question (PDF; DOCX)
Links to the Anchoring Concepts Content Map, differences in responses of students with and without a course in physical chemistry; codebook used to characterize student responses; examples of scientifically inaccurate ideas; examples of student representations of band structures (PDF)
