# Supplementary material for: How Do Inorganic Students and Instructors Describe Conductivity in Solids?
Source: J Chem Educ. 2026 Sep 9;103(10):5288–302. doi: 10.1021/acs.jchemed.5c01197 (PMC13674064; doi:10.1021/acs.jchemed.5c01197)

Supporting Information

How Do Inorganic Students and Instructors Describe Conductivity in Solids?

Barbara A. Reisner,[§*] Anne K. Bentley,[‡] Joanne L. Stewart,[#] Justin M. Pratt,[†] Shirley Lin,[‖] Jessie R. Hallers,[§] Jeffrey R. Raker,[⊥] Sheila R. Smith[▽]
*corresponding author: reisneba@jmu.edu

§ Department of Chemistry & Biochemistry, James Madison University, Harrisonburg, Virginia 22807, United States

‡ Department of Chemistry, Lewis & Clark College, Portland, Oregon 97219, United States

Department of Chemistry, Hope College, Holland, Michigan 49423, United States

† Department of Chemistry, University of Rhode Island, Kingston, Rhode Island 02881, United States

‖ Department of Chemistry, United States Naval Academy, Annapolis, Maryland 21402, United States

⊥ Department of Chemistry, University of South Florida, Tampa, Florida 33620, United States

▽ Department of Natural Sciences, University of Michigan—Dearborn, Dearborn, Michigan 48128, United States

Narrative Links to the Anchoring Concepts Content Map (p. 2)

Table S1 Comparison of Responses from Students Enrolled in Courses with and without a Physical Chemistry Prerequisite (pp. 2-3)

Table S2 Codebook with Full Example Responses (pp. 4-12)

Table S3 Examples of Scientifically Inaccurate Ideas in Student Responses (p. 13)

Figure S1 Visual Representations Included with Student Responses (p. 14)

Links to the Anchoring Concepts Content Map (ACCM). From our iterative qualitative analyses of student and instructor responses, four overarching categories were identified based on topic areas discussed: conductivity, doping, band structure, and temperature. It is interesting to note that these match the enduring understandings from the ACCM for inorganic chemistry, a consensus model developed by representative faculty member focus groups.(Marek ACCM) Our identified concepts appear across four anchoring concepts (or big ideas) in the ACCM: bonding (idea II); structure/function (idea III); experiments, measurement and data (idea IX); and visualization (idea X).

1. *The conductivity of solids can be explained by the band theory model. (*bonding: *II.G.2)*
2. *Many interesting properties of solids are due to the presence of defects or atoms on interstitial sites. (*structure/function: *III.H.3)*
3. *Temperature dependent conductivity and resistivity measurements are used to characterize metals, semiconductors, and superconductors. (experiments, measurement and data: IX.D.8)*
4. *Band theory is an extension of molecular orbital theory to the macroscale, and is used to explain electrical conductivity associated with metals, insulators, and semiconductors. (*visualization: *X.A.2)*

Table S1. Comparison of Responses from Students Enrolled in Courses with and without a Physical Chemistry Prerequisite

**Category**	**Idea**	**% of all students‡ *N* = 117**	**% students with no pchem prereq‡ *n* = 58**	**% students with pchem prereq‡ *n* = 59**	**Fisher's Exact Test *p* value**	**Benjamini-Hochberg Critical Value (rank)**
**conductivity**	C.0 no discussion of conductivity		20.7	3.4	0.0043*	0.0079 (3)**
	C.1 discuss any electron motion	56.4	41.4	71.2	0.0015*	0.0053 (2)**
	C.2 conductivity depends on temperature	57.3	53.4	61.0	0.4575	0.0421 (16)
	C.3 discuss any hole motion	24.8	19.0	30.5	0.1991	0.0237 (9)
**doping**	D.0 no discussion of doping	0.9	1.7	0.0	0.4957	0.0447 (17)
	D.1 classified, named, labeled	66.7	72.4	61.0	0.2402	0.0263 (10)
	D.2 conductivity changes with doping	77.8	74.1	81.4	0.381	0.0342 (13)

	D.3 new band is created by doping	32.5	25.9	39.0	0.1675	0.0211 (8)
	D.4 doping changes the number of charge carriers	56.4	51.7	61.0	0.3539	0.0316 (12)
**band structure**	BS.0 no discussion of band structure	25.6	32.8	18.6	0.0934	0.0184 (7)
	BS.1 band gap but nothing more	2.6	3.4	1.7	0.6185	0.0500 (19)
	BS.2 discuss charge carriers in relationship to band	53.0	39.7	66.1	0.0055*	0.0105 (4)**
	BS.3 changes in energy gap	37.6	41.4	33.9	0.4486	0.0368 (14)
	BS.4 use the language of molecules	17.1	6.9	27.1	0.0059*	0.0132 (5)**
	BS.5 any picture with bands	48.7	32.8	64.4	< 0.001**	0.0026 (1)**
**temperature**	T.0 no discussion of temperature	39.3	43.1	35.6	0.4521	0.0395 (15)
	T.1 any statement connecting temp and conductivity (how different from 1.2?)	58.1	55.2	61.0	0.5765	0.0474 (18)
	T.2 increased temperature promotes electrons	26.5	17.2	35.6	0.0355*	0.0158 (6)
	T.3 phonons disrupt conductivity	6.8	3.4	10.2	0.2719	0.0289 (11)

‡The numbers in each category do not add to 100%, because many respondents mentioned more than one idea.
* Indicates the difference between groups is significant at the $p < 0.05$ level.
** Indicates the difference between groups is significant based on the Benjamini-Hochberg procedure with a 5% false discovery rate.

Table S2. Codebook with Full Example Responses

Category	Full Description of Idea	Exemplars
**conductivity**	C.0 No discussion of conductivity.	"GaGe is an n-type semiconductor making the energy going from VB to CB a lot easier and more efficent [*sic*] that without doping Ge." *Student 7.125*<br><br>"The electronic structure of these two solids lead to differences in the observed conductivity by having one being doped and the other being undoped. Without Ge being doped, it acts as a conductor, but with it being doped with Ga, it turns into an insulator. Ga is in the 3rd period on the periodic table and in the 4th row, which means the size is bigger than Ge. Having an atom that is larger, will start to make the band gap smaller, until it is neglible [*sic*], which means it is an insulator." *Student 6.005*
	C.1 Discussion of any electron motion at all: *electrons jump or move between bands, orbitals, or atoms; being promoted; anything related to electron motion.*	"Ge doped with Ga is a p-type semiconductor, so the electrons stay in the valence band. When heated, **it's harder for the electrons to move, so conductivity decreases** with increasing temperature. When heated, the electrons in undoped Ge move to the conduction band easily, so conductivity increases with increasing temperature." *Student 7.104*<br><br>"When a germanium atom is doped with gallium, the fermi level is lowered closer to the valence band of the germanium atom; Increases in temperature **allows electrons to move from the valence band to the new band.** Thus creating holes in the valence band of Ge, which increases conductivity. The undoped Ge, there is large difference of energy which resists e- to move to the conduction band. Thus poor conductivity." *Student 2.006*
	C.2 Makes a connection between conductivity and temperature: *terms may be vague or respondents might say "heated"*.	"The undoped germanium has an increasing conductivity at higher temperatures than the germanium doped with gallium because germanium is larger than gallium. The larger germanium makes a smaller band gap,

		causing it to become a better conductor. The germanium with gallium starts out with more conductivity, but **as the temperature rises, the conductivity slowly decreases** because the band gap is slowly decreasing. Germanium also has an extra electron compared to gallium, which also affects the band gap." *Student 6.003*<br><br>"The gallium in the germanium allows holes to be created in the valence band. These holes allow greater conductivity than plain germanium. **As temperature increases the germanium conductivity increases** because it is easier for electrons to jump in the band gap. In the germanium doped with gallium the conductivity goes down w/ temperature increase because the electrons do not need to jump the band gap because the electrons can easily flow into the small band created by the gallium. So the increased excitement from the temperature reduces mobility decreasing conductivity in the germanium doped w/ gallium."<br>*Student 3.007*
	C.3 Discussion of any hole motion: *related to holes in bands.*	"Gallium has one less p electron than Germanium, therefore doping with Gallium will increase the conductivity significantly. This works because the energy gap between the highest and lowest energy orbitals (HOMO and LUMO) gets much smaller. Therefore, there are more "bands" that fill the conduction band. When the conduction band is filled, e- are able to be promoted to the HOMO, leaving "holes" in the LUMO. **The holes are able to hold a current**, which is why doping with gallium is more conductive."<br>*Student 5.010*<br><br>"Previous to any doping the conductivity itself is poor despite the increasing conductivity with temperature. The electronic structure is more in tune of a semiconductor that's adjustable w/ temperature. However, once the Ge is doped w/ gallium (Ga) the conductivity greatly increased despite its negative slope w/ temperature. This doping is in line w/ p doping which means **thres** [*sic*] **holes in the valence band to be able to circulate in the valence band**"<br>*Student 4.013*

**doping**	D.0 No discussion of doping.	"High conductivity in anode materials is necessary for transfer of electrons & Gallium greatly reduces thermal conductivity" *Student 7.135*
	D.1 One or both of the materials is / are classified, named, labeled: *as a p- or n-type semiconductor, conductor, metal, semiconductor - classification must be in the context of doping.*	"**It seems that undoped Ge is a semi-conductor and the Ge doped with Ga acts like a metal / conductor.** Semi-conductors conduct better at high temperature since more electrons can pass the band gap. Metals / conductors counduct better at low temperatures since the motion of the nuclei disrupt the electrons motion." *Student 4.010*<br><br>"**Undoped Germanium is an intrinsic semiconductor** thus have low conductivity, but with increase in temperature increases to some extent. However, **when Ge is dope with Ga it becomes an p-type semiconductor** which eventually gives an introduction of smaller energy gap in the band gap thus ultimately making Ge-Ga a better conductor due to easiness of flow of energy through the gap." *Student 7.129*
	D.2 Discussion of the fact that conductivity changes with doping: *discussion of conductivity differences before/after doping, not just one or the other.*	"**Ge doped w/ Ga has a much higher conductivity** b/c Ga cuts into the valence shell, making it easier for e- to go up to new block." *Student 2.005*<br><br>"Gallium has one less valence electron compared to germanium therefore making it a p-type dopant (hole-rich). **By decreasing electrons present in the molecule the conductivity increases** and stays more consistent as temperature is increased." *Student 3.011*
	D.3 Mentions that a new band is created by doping: *they can describe the band in words or represent it with a picture (has to be associated with Ga-doped Ge)*.	"**The doped Ge has an extra band from the trace amounts of Ga.** This allows the electrons to jump the gap more readily which results in a higher conductivity. The reason that the undoped Ge rises with temperature is because the heat energy allows the electrons to jump the gap more easily. The doped Ga declines slightly with heat because the heat is causing vibrations disturb the flow of electrons. It doesn't need the extra heat energy like undoped Ge so the heat doesn't help the doped Ga be more conductive." *Student 1.102*<br><br>"The germanium doped with gallium has a higher conductivity than undoped Ge

		because the addition of a p-type dopant creates "holes" or localized areas of less electron density. These holes make it easier for electrons to flow, resulting in higher conductivity. The inclusion of gallium **creates an additional band** lower the valence and conduction bands; this band is partially responsible for doped germanium's consistent conductivity across different temperatures, as additions to these holes/their band gap is not greatly affected by temperature. Undoped germanium is not very conductive at low temperatures due to a large band gap, but this band gap decreases with increasing temp. resulting in greater conductivity." *Student 5.012*
	D.4 doping changes the number of charge carriers (electrons or holes)	"**Since Ga has one less electron than Ge, it adds holes in the valence band.** That leads to higher conductivity than undoped Ge as seen in the above figure. Ga = 1 less e- = p-type" *Student 3.003*<br><br>"Because germanium (Ge) is a naturally occurring pure semiconductor it can be doped to increase or decrease its conductivity with the addition of other elements. The graph above shows that when Ge is doped with gallium (Ga) the conductivity increases, meaning that the band gap has been decreased resulting in an easier flow of electrons. Because of this observation it can be assumed that Ga results in a p-type conductivity because of its **deficit of electrons** in comparison to Ge. It can also be assumed that the radius of Ga is larger than that of Ge because of the band gap decrease observed." *Student 6.007*
**band structure**	BS.0 No discussion of band structure. *If this code is chosen, the response can at most have BS.5 as well.*	"Ge undoped has more electrons than if you were to replace some Ge with Ga. Thus GeGa is a p-type conductor." *Student 7.117*<br><br>"Undoped Ge acts as a semiconductor as its conductivity increases as the temperature increases. Ge that has been doped with Ga has a closer relation to that of a conductor due to the decrease in conductivity as the temperature increases. Another point that indicates that the undoped is a semiconductor while the doped is a conductor is based on the relitive [*sic*] conductivities of each as the doped is about 100 times more conductive than the

		undoped. The undoped may also be an insulator based on how low the conductivity is." *Student 6.102*
	BS.1 Discussion of the existence of a band gap but nothing beyond that. *If this code is chosen, the response can at most have BS.5 as well.*	"Ge doped with Gallium has a band gap that is compatible with UV. This increases the structure's absorption ability, thus increasing conductivity." *Student 7.109* [Factionless post 009]<br><br>"Since germanium has more electrons than gallium, when it is inserted, it will lead to a p-type doped conductor, since it is more like a general conductor and **the two bands** are so close, when it is heated it loses some conductivity." *Student 5.007*
	BS.2 Discussion of charge carriers in relationship to the band.	"Because germanium (Ge) is a naturally occurring pure semiconductor it can be doped to increase or decrease its conductivity with the addition of other elements. The graph above shows that when Ge is doped with gallium (Ga) the conductivity increases, **meaning that the band gap has been decreased resulting in an easier flow of electrons.** Because of this observation it can be assumed that Ga results in a p-type conductivity because of its deficit of electrons in comparison to Ge. It can also be assumed that the radius of Ga is larger than that of Ge because of the band gap decrease observed." *Student 6.007*<br><br>"In the Ge doped with Ga, as Ge has 4 valence e- and Ga has 3 valence e-, this is a p-doped semiconductor. As Ga has less valence e- than Ge, **there are holes left in the valence band. This allows e- to move around more freely in the valence band (in comparison with undoped Ge), as e- can shift to fill those holes.** Therefore Ge doped with Ga has a higher observed conductivity than undoped Ge. As the Ge is heated, the e- are excited up into the conduction band, leading to higher conductivity." *Student 4.011*
	BS.3 Discussion of changes in the energy gap such as size of elements affecting band gap size, effect of temperature on band gap, effect of extra electrons on band gap. *This code is not selected if the student only said a new band is created, as that is code D3.*	"By doping the Ge with Ga (p-type doping here), **there is an effective reduction in the band gap/E needed to conduct electricity** by adding in a series of holes associated with Ga. This reduction in required energy leads to the higher conductivity of the doped solid."

		Student 5.003<br><br>"Because Ga's empty orbital is energetically between the filled and empty orbital for Ge. This **decreases the delta E required to excite e- to an empty orbital (deltaE2 < deltaE1).** Because it takes less energy to excite the electrons, the doped Ge conducts better than the undoped Ge. (The doped Ge would be a p-type conductor.)" *Student 2.003*
	BS.4 Uses the language of molecules such as orbitals, HOMO/LUMO, references to *s* and *p* orbitals.	"Ge doped with Ga is a better semiconductor because **the Ga impurities decrease the energy level between the s-orbitals and p-orbitals** allowing the e- to move b/t orbitals. A lower temperature is needed to promote e- into the next energy level. As the graph shows, w/o the Ga present, higher temps are needed to promote e- from the s-orbital (arrow) p-orbital. [Also provides electron configuration] Ge [Ar] 4s2 4p2 Ga [Ar] 4s2 4p1" *Student 1.001*<br><br>"Gallium has one less p electron than Germanium, therefore doping with Gallium will increase the conductivity significantly. This works because the energy gap between the **highest and lowest energy orbitals (HOMO and LUMO)** gets much smaller. Therefore, there are more "bands" that fill the conduction band. When the conduction band is filled, e- are able to be promoted to the HOMO, leaving "holes" in the LUMO. The holes are able to hold a current, which is why doping with gallium is more conductive." *Student 5.010*
	BS.5 Any picture / sketch that shows something about bands: *the drawing must be clearly associated with bands.*	Student sketches that did satisfy idea BS.5:<br><br>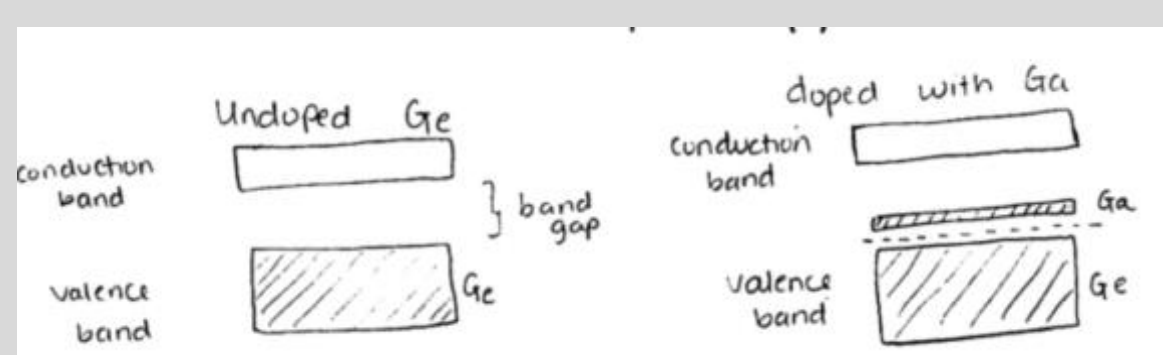<br>*Student 2.006*

		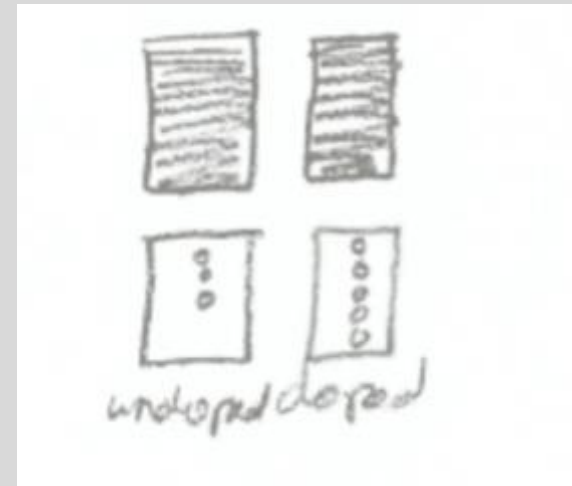<br><br>*Student 7.017*<br><br>Student sketches that did *not* satisfy idea BS.5:<br><br>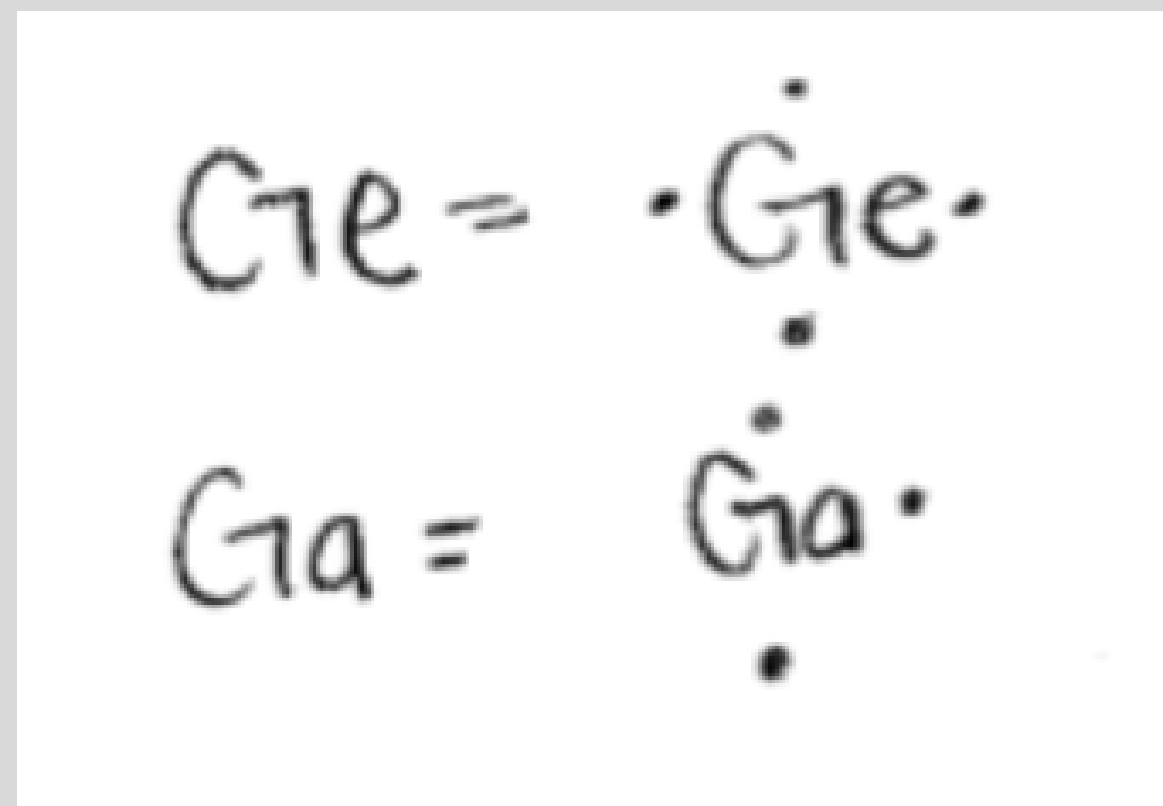<br><br>*Student 2.002*
**temperature**	T.0 No discussion of temperature.	"Gallium is a p-type dopant for Germanium enusing [*sic*] holes in the valence band resulting in higher conductivity."<br>*Student 3.001*<br><br>"The gallium having only 3e- creates a hole where the fourth bond would be"<br>*Student 2.103*
	T.1 General statement that connects temperature and conductivity data.	"undoped Ge increases conductivity with an increase in temperature which is a not a common charcteristic [*sic*] for metals. **Metal conduct worse in high temps.** When Ge is doped with Ga the conductivity starts to slowly decrease with the increase in temperature. Ge & Ga together have characteristic of a semiconductor where conductivity is better at colder temperatures."<br>*Student 4.005*<br><br>"Ge on its own is a semiconductor. Ga would serve as a p-type to Ge causing more holes in the current thus evening out the charge. **The temperature on the graph indicates that Ge by itself would be more**

		**conductive** on its own whereas the Ge doped with Ga would be less conductive but providing a more even current." *Student 3.005*<br><br>"Ge doped w/ gallium is a p-type semiconductor this would slightly decrease the conductivity due to the band gap. The undoped Ge has a consistent band gap that isnt disrupted by the addition/elimination (GeGa) of e- allowing **conductivity to increase w/ temperature**" *Student 7.108*
	T.2 Discussion of how increased temperature promotes electrons to higher energy levels or across the band gap.	"The undoped Ge has a larger band gap than the Ge doped w/ Ga. Ga is a p-type dopant to Ge because it adds an acceptor level close to the donor level that allows for better transfer of electrons which increases conduction. **As temp. increases the undoped Ge is able to excite more e- to the conduction band increasing conductivity** but this doesn't really affect doped Ge." *Student 3.004*<br><br>"Ge doped with Ga created a p-type semiconductor, while undoped Ge is an intrinsic semiconductor. While doped Ge has a lower band gap and greater initial conductivity, it will drop in conductivity as temperature increases due to phonons. Undoped Ge has a larger initial band gap, but **as temperature increases will greatly increase in conductivity due to electrons being promoted** and not facing the same phonon interferance [*sic*] as the crystalline doped Ge." *Student 7.114*
	T.3 Discussion of how or why increased temperature leads to a decrease in conductivity for metals: ie, via dissipation of electron energy through phonon vibrations.	"The doped Ge has an extra band from the trace amounts of Ga. This allows the electrons to jump the gap more readily which results in a higher conductivity. The reason that the undoped Ge rises with temperature is because the heat energy allows the electrons to jump the gap more easily. **The doped Ga declines slightly with heat because the heat is causing vibrations disturb** [*sic*] **the flow of electrons.** It doesn't need the extra heat energy like undoped Ge so the heat doesn't help the doped Ga be more conductive." *Student 1.002*<br><br>"It seems that undoped Ge is a semi-conductor and the Ge doped with Ga acts

		like a metal / conductor. Semi-conductors conduct better at high temperature since more electrons can pass the band gap. **Metals / conductors counduct better at low temperatures since the motion of the nuclei disrupt the electrons motion.**" *Student 4.010*

Table S3. Examples of Scientifically Inaccurate Ideas in Student Responses

**Idiosyncratic Idea**	**Student Quote**
1. Changing the temperature changes the band gap.	Undoped germanium's conductivity decreased with increasing temperature, acting as a conductor due to its band gap decreasing as the kinetic energy of the atoms increased. *Student 6.008*
1. The number of electrons, not the occupancy of states, changes conductivity.	Ge doped w/ Ga is a p-type. This is why there is a decrease in conductivity. However Ge by itself has more electrons therefore is better conductor as temp is increase. *Student 7.115*
1. Conductivity depends on time.	We should expect to see the doped semi conductor to fall slightly while it garners the energy to overcome its band gap, then rise. It has a higher conductivity at the start because e- were able to cross quickly. *Student 7.132*
1. Atomic size is responsible for changing the conductivity.	The undoped germanium has an increasing conductivity at higher temperatures than the germanium doped with gallium because germanium is larger than gallium. *Student 6.003*

Figure S1. Visual Representations Included with Student Responses

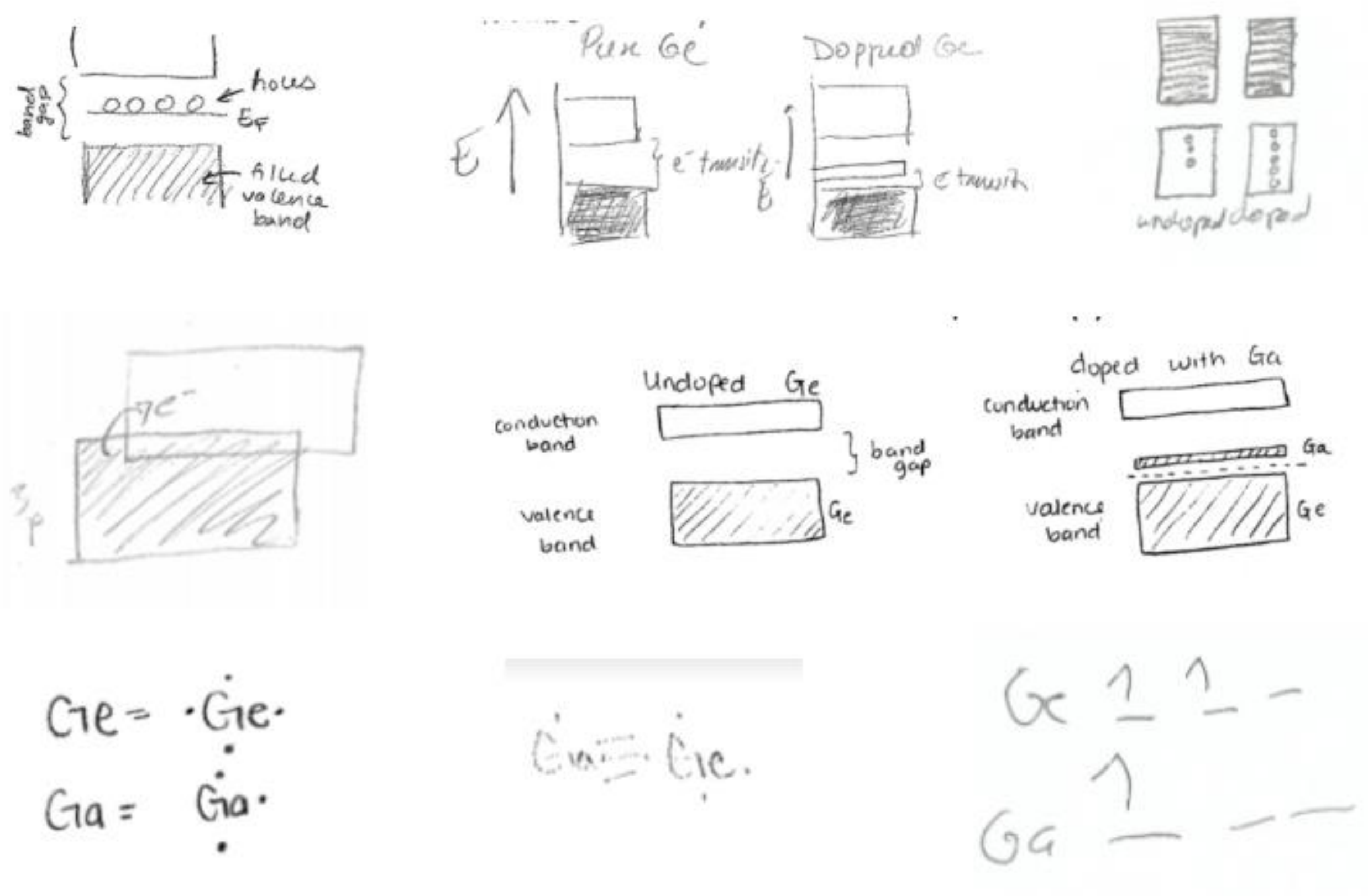

Supplement: Supplementary file 2 [file ed5c01197_si_003.pdf]
