# Supplementary material for: How Do Inorganic Students and Instructors Describe Conductivity in Solids?
Source: J Chem Educ. 2026 Sep 9;103(10):5288–302. doi: 10.1021/acs.jchemed.5c01197 (PMC13674064; doi:10.1021/acs.jchemed.5c01197)
Supplement: Supplementary file 3 [file ed5c01197_si_002.docx]

**Supporting Information – Conceptual Question**

**How Do Inorganic Students and Instructors Describe Conductivity in Solids?**

Barbara A. Reisner,^§^* Anne K. Bentley,^‡^ Joanne L. Stewart,^#^ Justin M. Pratt,^†^ Shirley Lin,^∥^ Jessie R. Hallers,^§^ Jeffrey R. Raker,^⊥^ Sheila R. Smith^∇^

*corresponding author: [reisneba@jmu.edu](mailto:reisneba@jmu.edu)

§	Department of Chemistry & Biochemistry, James Madison University, Harrisonburg, Virginia 22807, United States

‡	Department of Chemistry, Lewis & Clark College, Portland, Oregon 97219, United States

\#	Department of Chemistry, Hope College, Holland, Michigan 49423, United States

†	Department of Chemistry, University of Rhode Island, Kingston, Rhode Island 02881, United States

∥	Department of Chemistry, United States Naval Academy, Annapolis, Maryland 21402, United States

⊥	Department of Chemistry, University of South Florida, Tampa, Florida 33620, United States

∇	Department of Natural Sciences, University of Michigan—Dearborn, Dearborn, Michigan 48128, United States

Below is a graph of the conductivity as a function of temperature for undoped germanium (Ge), and germanium highly doped with gallium (Ga). Using complete sentences and drawing any additional diagrams or graphs you need, describe how the electronic structure of these two solids leads to differences in the observed conductivity.
